# Thiazolidinedione-Based PI3Kα Inhibitors: An Analysis of Biochemical and Virtual Screening Methods

**DOI:** 10.1002/cmdc.201000467

**Published:** 2011-01-04

**Authors:** Jo-Anne Pinson, Oleg Schmidt-Kittler, Jiuxiang Zhu, Ian G Jennings, Kenneth W Kinzler, Bert Vogelstein, David K Chalmers, Philip E Thompson

**Affiliations:** a Medicinal Chemistry & Drug Action, Monash Institute of Pharmaceutical SciencesParkville, Victoria 3052 (Australia); b The Ludwig Center for Cancer Genetics and Therapeutics and the Howard Hughes Medical Institute at the Kimmel–Hopkins Cancer CenterBaltimore, MD 21231 (USA)

**Keywords:** homology modeling, inhibitors, PI3K kinases, rhodanine, thiazolidinedione

## Abstract

A series of synthesized and commercially available compounds were assessed against PI3Kα for in vitro inhibitory activity and the results compared to binding calculated in silico. Using published crystal structures of PI3Kγ and PI3Kδ co-crystallized with inhibitors as a template, docking was able to identify the majority of potent inhibitors from a decoy set of 1000 compounds. On the other hand, PI3Kα in the apo-form, modeled by induced fit docking, or built as a homology model gave only poor results. A PI3Kα homology model derived from a ligand-bound PI3Kδ crystal structure was developed that has a good ability to identify active compounds. The docking results identified binding poses for active compounds that differ from those identified to date and can contribute to our understanding of structure–activity relationships for PI3K inhibitors.

## Introduction

The class I phosphatidylinositol 3 kinases (PI3Ks) are a family of four iso-enzymes (PI3Kα, β, γ and δ) that catalyze phosphorylation of phosphatidylinositides (PI) to form second messenger lipids, which regulate numerous cellular functions including cell growth, motility, proliferation and survival.^1^, ^2^ PI3K inhibitors are currently targets for therapeutic application in a range of diseases including cancer,^3^, ^4^ thrombosis,^5^ and immunoinflammatory disease.^6^, ^7^ Numerous PI3K inhibitors have been described in recent years, and some show isoform selectivity.^8^, ^9^ The development of these inhibitors can be traced from hit compounds identified by screening libraries followed by medicinal chemistry campaigns. Filters for expediting the discovery of hits or leads can have many guises, including druggability, comparative association with known structural motifs (a focused library) and in silico assessment. Herein, we have examined the latter two filters to obtain information about inhibitors of PI3 kinase. With crystal structures of PI3K isoforms including co-crystallized ligands and apo-structures deposited in the Protein Data Bank (PDB), virtual screening is an attractive possible filter. Such methods offer the opportunity to minimize the wet laboratory effort.

Molecular docking studies have been used in a number of contexts by other workers, but have typically been used to give post-hoc explanation of the potency of selected compounds. Earlier studies were restricted to PI3Kγ or homology models derived from PI3Kγ.^10^–^18^ For example, Pomel et al. described the use of induced fit docking experiments in the development of furan-2-ylmethylene thiazolidinediones as potent inhibitors of PI3Kγ.^19^ Other studies utilized homology models of PI3Kα based on PI3Kγ and mTOR to optimize series of 4-imidazolopyrimidines and morpholinopyrrolopyrimidines (PDB: 3IBE).^15^, ^17^ Virtual screening experiments have predicted novel scaffolds for optimization in the context of both pan and isoform selective inhibition of PI3K.^20^, ^21^

Given the recent release of the co-ordinates for PI3Kα (2RD0) and PI3K*δ* (2WXL), attention has turned to docking studies specific to those biochemical targets. Use of molecular docking in PI3Kα has been targeted, in particular, at the study of PIK75, a potent and α-selective inhibitor. Models of binding that explain PIK75 selectivity have been proposed by Denny and Frederick et al., and Han and Zhang using docking models based upon PI3Kγ.^22^, ^23^ More recently, Sabbah et al. extended the docking study of this class to 13 active analogues as well as other chemotypes.^24^ These more recent studies have also used molecular dynamics simulations as part of the docking procedures.

As more crystallographic data becomes available, the success of these models can be more directly assessed. Notably, the crystal structure of ZSTK474^25^ shows the ligand in a very different pose to that predicted by modeling.^26^ In other cases, the scoring functions of molecular docking have been unable to explain observed ligand binding affinities.^27^

The sum total of these studies does not give a clear picture of the best approach to implementing virtual screening for PI3K inhibitors. Our aim has been to develop a robust process for virtual screening for PI3Kα inhibitors, which gives a good enrichment of actives out of compound sets, and we were particularly attracted to the study of thiazolidinedione-based compounds.

Among these thiazolidinedione compounds, AS-604850 (**1**) and AS-605240 (**2**) are selective inhibitors of PI3Kγ and show anti-inflammatory activity in animal models of chronic inflammation.^7^, ^28^ They were also successfully co-crystallized with PI3Kγ. Compound **2** also shows potent inhibition of the PI3Kα isoform, and as such the thiazolidinedione class could also be considered a starting point for the design of selective PI3Kα inhibitors.^14^ Molecular docking studies covering a broad series of this structural class against PI3K have not yet been reported.

Thiazolidinediones and their sulfur analogues, rhodanines, are also well suited to evaluation by in vitro screening methods as they are widely available from commercial sources or can be accessed by straightforward syntheses.^28^–^30^ We therefore have had the opportunity to assess the results of virtual screening experiments conducted against multiple enzyme models in comparison to biochemical screening assay data for over 70 compounds. While we identified diverse compounds that displayed both sub-micromolar PI3Kα potency and isoform selectivity from the screens, the comparison of the approaches allowed us to find the most effective model for retrieving our active compounds from the decoy set. That turned out to be a PI3K*δ* structure, which has been solved to good resolution and co-crystallized with the pan-PI3K inhibitor ZSTK474. Models of the PI3Kα structure, from the crystal structure, were unable to produce useful enrichment from a library of decoys. However, a homology model of PI3Kα derived from PI3K*δ* and utilizing induced fit docking did give improved results. The influence of parameters such as protein structure homology, resolution and binding site occupancy is of significance both in the context of continuing PI3K inhibitor discovery and also the numerous other targets of this compound class.

## Results and Discussion

### Compound selection, synthesis and structure– activity relationships of thiazolidinedione derivatives as PI3K isoform inhibitors

**Figure 1:**
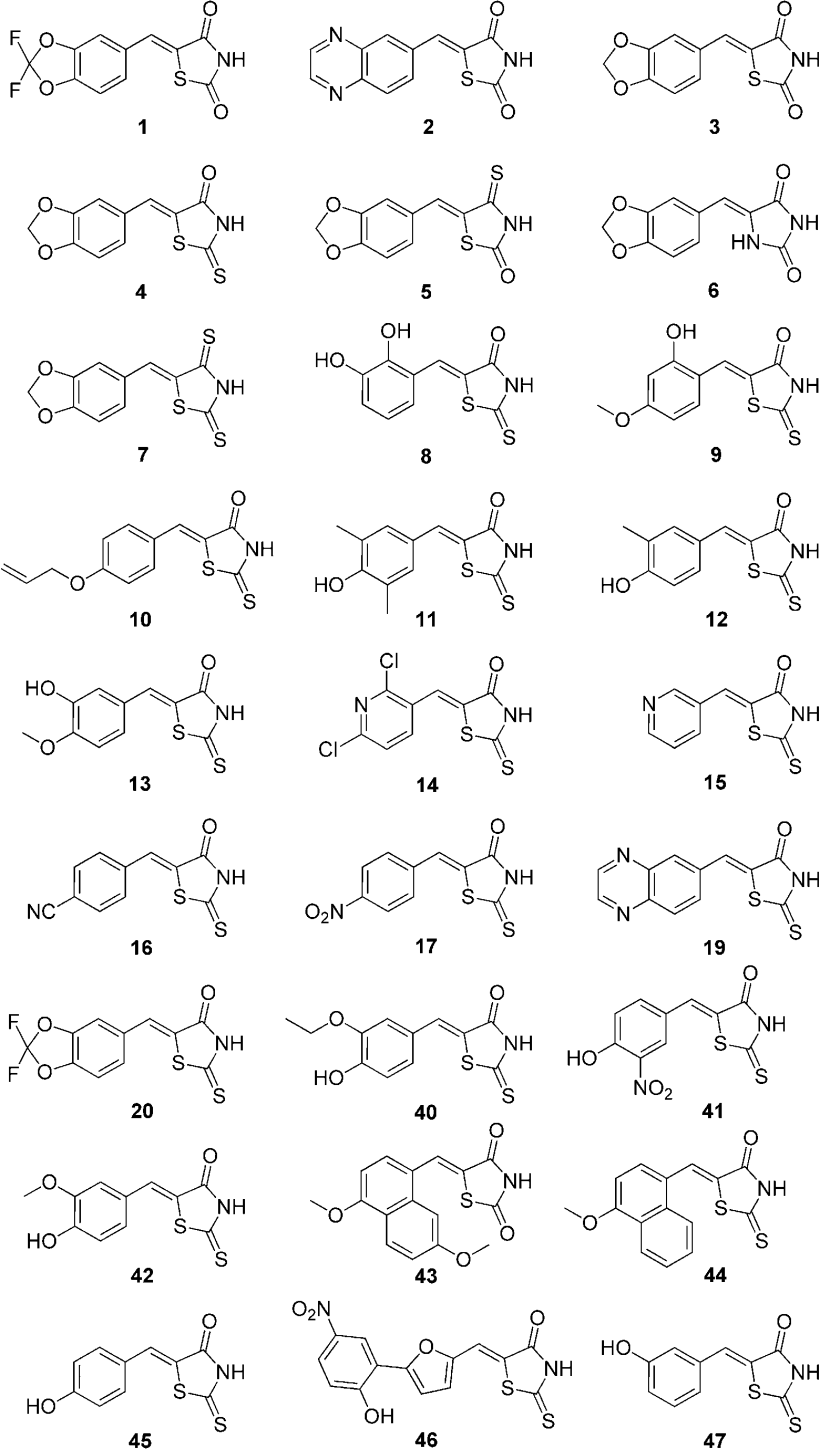
The structures of compounds 1–17, 19–20, and 40–47 discussed in the text.

The chemical and biochemical data is presented first for clarity. Compounds were chosen based upon structural comparison to the compounds **1** and **2**, and ready availability either from commercial sources for immediate assay, or by Knoevenagel condensation from precursor aldehydes.^28^–^30^ (Figure 1, figure S1 in the Supporting Information). Compounds with substituents on the thiazolidinedione or rhodanine ring were excluded from this study. Seventy-three derivatives were screened as inhibitors of recombinant PI3Kα and PI3Kγ using an in vitro recombinant PI3K assay as previously reported.^31^, ^32^

**Figure 2:**
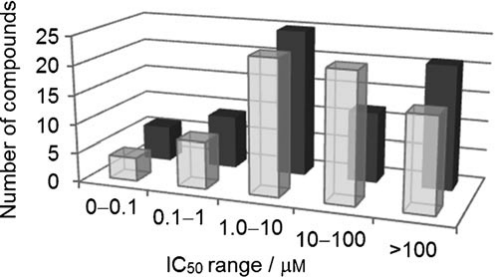
Number of thiazolidinedione scaffolds against PI3Kα (▪) and PI3Kγ (▪) in a given activity range (μm).

The results of the screening assays are shown in Figure 2 and Table 1. We were able to confirm the reported IC_50_ values of AS-604850 (**1**) and AS-605240 (**2**).^7^ Nearly half of the compounds tested showed an IC_50_ value of less than 10 μm, but the full series shows inhibitor potency spanning five orders of magnitude highlighting that the compound set should provide a useful test to molecular docking experiments.

**Table 1:** IC_50_ values of selected compounds against PI3Kα and PI3Kγ.

Compd	IC_50_ [μm]	Ratio
	PI3Kα	PI3Kγ	α/γ
**1**	4.5	0.30	18
**2**	0.060	0.0080	7.5
**3**	0.050	0.040	1.3
**4**	0.25	0.10	2.5
**5**	0.45	0.12	3.8
**6**	50	>100	<0.50
**7**	1.9	1.0	1.9
**8**	7.3	27	0.30
**9**	4.4	9.3	0.50
**10**	2.7	4.9	0.60
**11**	0.069	0.042	1.6
**12**	0.15	0.12	1.2
**13**	2.7	0.25	11
**14**	11	1.5	7.1
**15**	9.4	3.1	3.1
**16**	86	4.0	22
**17**	22	1.4	16
**19**	0.0030	0.0013	2.2
**20**	8.3	0.62	13
**40**	3.0	0.41	7.3
**41**	11	1.4	7.9
**42**	0.14	0.060	2.3
**43**	8.7	>100	<0.087
**44**	9.0	>100	<0.090
**45**	0.40	0.20	2.0
**46**	0.10	0.025	4.0
**47**	0.80	1.0	0.80

Twelve compounds were found to have a sub-micromolar IC_50_ value against PI3Kα, and fifteen against PI3Kγ. The IC_50_ values of the most potent compounds against PI3Kα and PI3Kγ are listed in Table 1. The majority of these compounds showed no particular preference for either of the isoforms (figure S2 and table S1 in the Supporting Information). Seven compounds (**13**, **14**, **16**, **17**, **20**, **40** and **41**) demonstrated selectivity for PI3Kγ (α/γ ratio range 7–21 fold). Some compounds (**8**, **9**, **10**, **43** and **44**) exhibited preference for the α-isoform, but they were of moderate potency. The remainder were neither particularly potent nor selective (table S1 in the Supporting Information).

It was observed that a number of structurally similar compounds showed different potencies against PI3Kα or PI3Kγ. Most obviously, the difference between **1** and **3** was the methylene replacement of the difluoromethylene group. Compound **3** is a moderately potent and nonselective inhibitor of PI3Ks and also inhibits PI3Kβ potently (data not shown). The inclusion of fluorine atoms into the dioxole ring clearly plays a central part in developing the PI3Kγ selectivity of **1**, but this largely derives from a major loss of potency against PI3Kα. Interestingly, we found a similar induction of PI3Kγ selectivity for analogues of the nonselective compound **42**, which has a 3-methoxy-4-hydroxyaryl arrangement. In compound **13** these substituents are interchanged, but this compound is nearly 20-fold less potent against PI3Kα. Similarly, compound **40**, which differs from **42** only by replacement of the methoxy substituent with an ethoxy, shows a reduced ability to inhibit PI3Kα.

We also investigated modification of thiazolidinedione by replacing oxygen with sulfur at the 2- and/or 4-positions. We tested a number of compounds derived from piperonal (**3**, **4**, **5**, **6**, **7**) and found that thiazolidinedione **3**, rhodanine **4** and isorhodanine **5** compounds were comparable in both selectivity and potency (Table 1). On the other hand, rhodanine compound **19** showed very potent activity, nearly 20-fold more potent at PI3Kα than the thiazolidinedione counterpart **2**. The thiorhodanine derivative **7** was 10-fold less active at both isoforms, and the hydantoin equivalent **6** was also a poor inhibitor of both isoforms. This suggests that change in size and electron-density distribution of thiorhodanine or hydantoin groups^33^, ^34^ does impact on binding to the catalytic site of PI3Kα. The same pattern was also found to be true of PI3Kβ and PI3K*δ* (data not shown). Finally, compounds **11** and **12** differ only by the methyl substituent in the 5-position. This group yielded a threefold improvement in potency, implying an additional hydrophobic interaction within the catalytic site.

**Figure 3:**
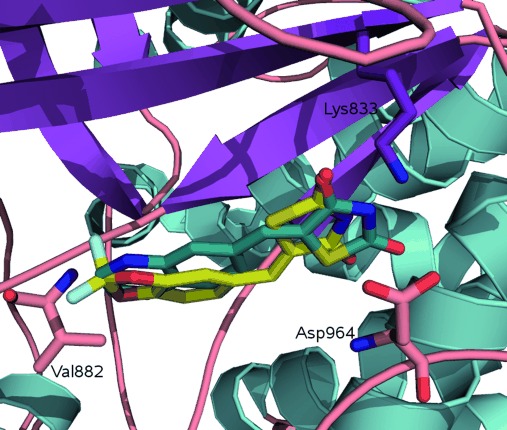
Co-crystallized structures of: AS-604850 (1, PDB: 2A4Z; yellow) and AS-605240 (2, PDB: 2A5U; blue) showing dual binding modes. These compounds are tethered by making a hydrogen-bonding interaction with Val 882 and a salt bridge with Lys 833.

In most cases, the potency of compounds was consistent with the picture of ligand binding derived from the reported X-ray structures. Within the binding site of PI3Kγ, the 1,3-benzodioxole oxygen of **1** and quinoxaline nitrogen of **2** form a hydrogen bond with the Val 882 amide backbone. The thiazolidinedione nitrogen interacts with Lys 833 via a salt-bridge interaction or hydrogen-bonding interactions with one or both of Lys 833 and Asp 964 (PI3Kγ). These residues are conserved in PI3Kα, and the active inhibitors in general appear capable of matching those requirements. Interestingly, compounds **1** and **2** were shown to adopt different poses in the PI3Kγ crystal, flipped through 180° (Figure 3), demonstrating that compounds of that class have at least two orientations in the binding site, but there is no evidence of significant ligand-induced enzyme side-chain perturbations.

However, some of our identified inhibitors would not be expected to fit with either of these binding poses. The 3-pyridyl derivatives **14** and **15** appear to be unable to span the binding site between the critical hinge residue, Val 882 and the salt bridge of Lys 833–Asp 964. Similarly, compounds **16** and **17** do not appear capable of making comparable interactions to those observed in X-ray structures suggesting that they might adopt different binding site poses related to these substitutions.

### Virtual screening

The crystal structures of compounds **1** and **2** with PI3Kγ (Figure 3) provide a template for understanding the inhibitor-enzyme interactions.^7^ Analogous interactions are observed in most PI3K inhibitor complexes.^7^, ^35^–^38^ It has been shown that PI3K isoforms cluster in their sensitivity to certain inhibitors^37^ such that compounds potent at PI3Kβ tend to inhibit PI3K*δ* more readily than other isoforms. Similarly, compounds effective at PI3Kα also inhibit PI3Kγ.^37^ This is supported by sequence homology across the isoforms with the β and *δ* most closely related,^1^, ^36^, ^39^, ^40^ particularly proximal to the ATP binding site.

In virtual screening of the compound set, we analyzed the influence of a series of parameters defined by protein structure. Naively, it might be thought that docking the compound set against the crystal structure of PI3Kα (PDB: 2RD0)^4^ would be the most relevant choice. However, that structure is of the apo-enzyme form and is only resolved to 3.0 Å. On the other hand, the PI3Kγ structure (PDB: 2A5U, 2.7 Å)^7^ might be an excellent model for this study as it is “pre-organized” as a co-crystal with a thiazolidinedione ligand, yet the structural resolution is relatively modest. One of the highest resolution PI3K structures available to us was PI3K*δ* (PDB: 2WXL, 1.99 Å) co-crystallized with ZSTK474.^25^ This is also a ligand-bound structure, with key interactions involving conserved binding site residues, consistent with the nonselective nature of the inhibitor.

The compound set was docked, in an approach similar to that described by McRobb et al.,^41^ using a set of 1000 drug-like decoy compounds^42^ (available from the Schrödinger web site) into the available X-ray crystal structures and derived models. The use of decoy sets provides a useful measure of the discriminatory power of a docking process, measuring the ability of the docking procedure to identify active compounds early from an unbiased series.^43^, ^44^ Compounds were built and minimized in Sybyl,^45^ then prepared using LigPrep.^46^ Virtual screening was performed using GLIDE 5.5 or 5.6 (Schrödinger)^47^–^49^ extra-precision (XP) mode with rigid receptor. All rhodanine derivatives were modeled with both protonated and deprotonated nitrogens^50^ as the p*K*_a_ of this group is predicted to be between 6.42 and 8.44 (average of 7.44) using Advanced Chemistry Development Inc. software (table S2 in the Supporting Information).^51^ Compounds with an IC_50_ value for PI3Kα of 50 μm or less were defined as active. The decoy set, enriched with 52 active compounds, was docked into each model and ranked by GlideScore to obtain one pose per ligand (tables S3 and S4 in the Supporting Information). The abundance of active compounds relative to decoy compounds in these rankings was then assessed using receiver operating characteristic (ROC) curves.^52^

**Figure 4:**
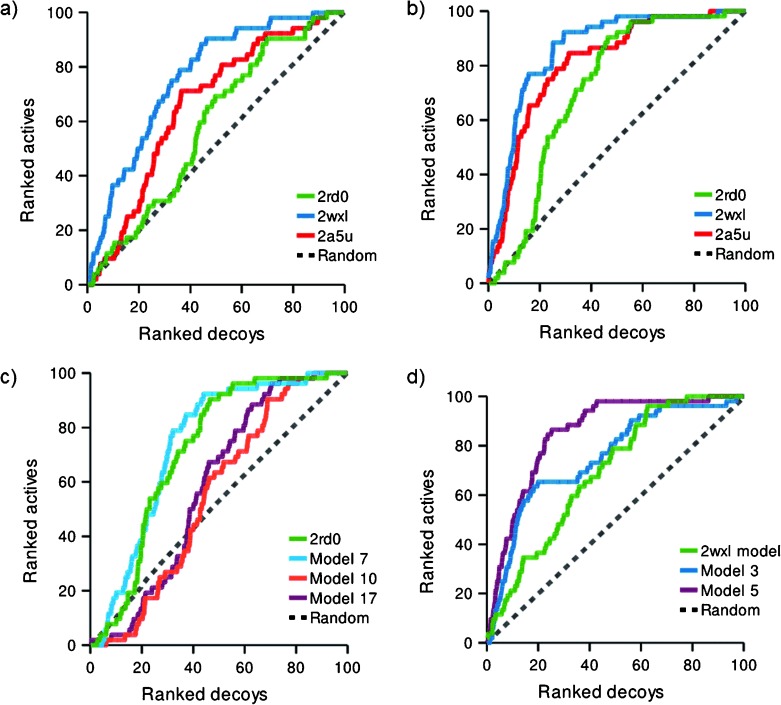
ROC curves for docked ligands and decoy compounds based upon PI3Kα IC_50_ data. The dotted line shows the expected result if there is no enrichment. a) Deprotonated and b) protonated ligands docked into PI3K crystal structures: PI3Kα (2RD0), PI3Kγ (2A5U) and PI3K*δ* (2WXL). c) Docking into induced fit poses generated from the apo-PI3Kα structure 2RD0 using protonated ligands. d) Docking into induced fit poses for PI3Kα homology models derived from the ligand-bound PI3K*δ* structure (2WXL) using protonated ligands.

**Figure 5:**
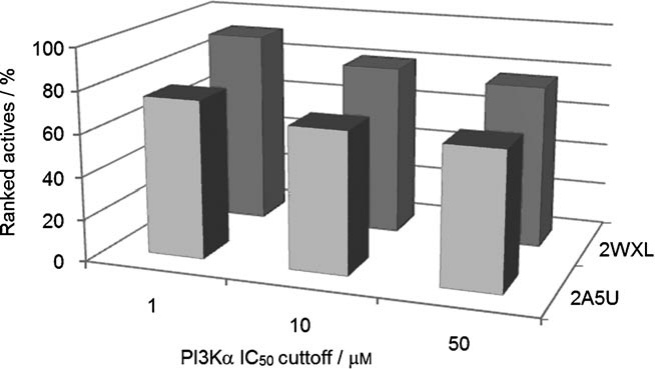
Summary of identified active protonated compounds within the top 20 % of the library for both the 2A5U (▪) and 2WXL (▪) crystal structures at cutoffs of 1, 10 and 50 μm for PI3Kα.

Figures 4 a and 4 b show ROC curves for the docking of ligands in either the deprotonated or protonated states into the PI3Kα (2RD0), PI3Kγ (2A5U) and PI3K*δ* (2WXL) crystal structures. Immediately apparent is the poor performance of the virtual screen in docking ligands into PI3Kα. Fundamentally, there is no preferential selection of active compounds in the top 20 %, and this is true irrespective of the chosen protonation state of the ligand set. On the other hand, the docking results for the chosen PI3Kγ and PI3K*δ* structures show prominent enrichment of the test compounds from the decoy set. Docking protonated ligands (Figure 4 b, Table 2), 65 % and 77 % of the active compounds were retrieved from the top 20 % of the library, respectively. The ionization state of the library was found to have a marked influence on these results with the protonated series more successfully retrieved. In all subsequent analyses, only results from the protonated series are considered. Virtually identical curves were obtained when PI3Kγ IC_50_-based ranking was used (data not shown), not surprising given the strong correlation of PI3Kα and PI3Kγ inhibition. One other parameter that we assessed was the arbitrary definition of active compounds as IC_50_ <50 μm, which might be considered a generous cutoff. Interestingly, changing this cutoff to a more stringent test at 10 μm or 1 μm (Figure 5) resulted in an even better selection of ranked actives for PI3Kα, for both 2A5U and 2WXL. Docking into 2WXL, 11 of the 12 sub-micromolar inhibitors were retrieved in the top 20 % of the library.

**Table 2:** Summary table of G-Score, ROC and Enrichment at 20 % for protonated TZD ligands.

Structure	G-Score		G-Score range		ROC		Enrichment	
	Mean	Median	Lower limit	Upper limit	[AUC]	at 20 % [%]
2A5U	−5.59	−5.69	−10.14	−3.75	0.849	65.4
2WXL	−5.85	−5.96	−11.73	−3.57	0.912	76.9
2RD0	−5.39	−5.46	−8.31	−2.96	0.748	23.1
IFM7^[a]^	−7.15	−7.14	−9.57	−5.82	0.772	32.7
α-model (2WXL)	−8.46	−8.61	−13.69	−7.13	0.721	34.6
IFM3^[a]^	−7.82	−8.03	−12.41	−3.84	0.788	61.5
IFM5^[a]^	−8.54	−8.62	−12.35	−6.67	0.900	65.4

[a] IFM: induced fit-derived model.

The results show the clear superiority of PI3Kγ and PI3K*δ* crystal structures for docking in comparison to the PI3Kα structure. The fact that the target isoform PI3Kα was a poor template for screening these compounds compared to PI3Kγ and PI3K*δ* was somewhat surprising. The major differences would appear to be the use of a ligand-templated crystal structure for the PI3Kγ and PI3K*δ* study, but the improved resolution of the PI3Kγ and PI3K*δ* crystal structures may also have played a significant part in determining the quality of the docking solutions, as seen elsewhere.^53^

**Figure 6:**
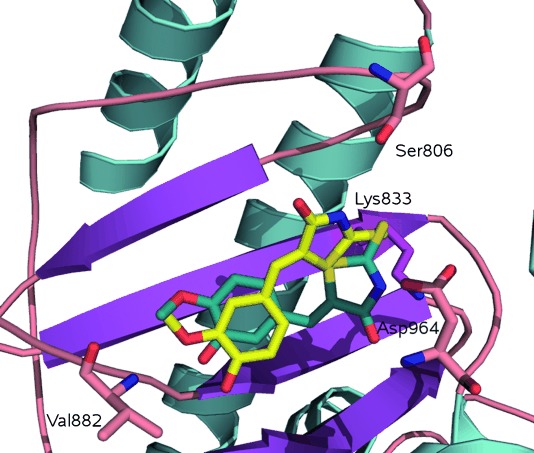
Alternate predicted docking poses of **13** (blue) and **42** (yellow) [protonated] in PI3Kγ showing the hydroxy or methoxy interacting with the amide backbone of Val 882, while the thiazolidinedione nitrogen of **13** and **42** interacts with the side chain of Lys 833 or Ser 806 respectively.

Our biochemical screen identified some subtle but significant influences brought by structural modification, and not explained by a simple pharmacophore model based on the existing co-crystals with PI3Kγ. As described above, inspection of our docking results showed that, as well as the two observed crystal poses for thiazolidinediones, alternate binding site poses were identified in compounds that proved to have high potency. In particular, this may have impacted on the observation of PI3Kγ selectivity. Compounds with a *para*-hydroxy substituent (**11**, **12**, **16**, **40**, **41**, **42** and **45**) adopted a binding site pose in PI3Kγ (2A5U) where the interaction with Val 882 was maintained, but the thiazolidinedione rotated away from the Lys 833–Asp 964 pairing, making an unprecedented contact with Ser 806. This may be significant, as five of the six compounds are sub-micromolar inhibitors. Isomers **13** and **42** are able to overlay their catechol monoether portion very closely, but project the rhodanine ring in different directions (Figure 6). This may explain why **13** (PI3Kγ-selective) and **42** (nonselective) display different isoform selectivities.

**Figure 7:**
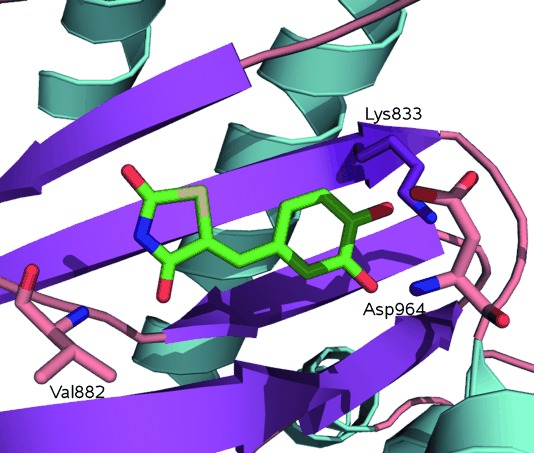
Alternate predicted docking pose showing catechol of **56** [protonated] interacting with side chain of Lys 833, and thiazolidinedione with the amide backbone of Val 882 in PI3Kγ.

The pyridyl derivatives **14** and **15** also showed interesting docking solutions. These smaller compounds docked in the expected orientation, but the distances to both Val 882 and Lys 833–Asp 964 were over 3.0 Å, consistent with the moderate potency. Finally, in some cases, compounds docked “back-to-front” such that the thiazolidinedione or rhodanine moiety interacted with the amide backbone of Val 882, while substituents on the aryl ring formed hydrogen bonding interactions with the side chain of Lys 833 (Figure 7). While clearly these results are open to interpretation and can only be supported by crystallographic evidence, it has been shown that multiple conformations of a particular ligand within the same protein can exist.^54^, ^55^ Importantly, in considering structural elaboration based on any of these hits, the possibility of multiple binding modes within a compound class could provide the medicinal chemist with alternate pathways to optimized compounds.

In attempting to rationalize the poor results obtained using the PI3Kα crystal structure, we wondered if the apo state of the enzyme crystal structure was the key contributing factor and whether this might be overcome, either by the refinement of the crystal structure using induced fit docking or by the development of a homology model derived from a liganded homologue, such as PI3Kδ. Standard docking methods hold the receptor binding site rigid, which is not a true representation of the dynamic state of the protein. Methods such as molecular dynamics simulations, though computationally expensive, model continuous protein motion,^56^ where frames can be used in virtual screening experiments. This process can be simulated by induced fit docking, which allows for ligand and protein flexibility.^41^, ^57^, ^58^ To improve the recognition of active compounds in the PI3Kα model (2RD0), we used induced fit docking with Glide 5.6 and Prime 2.2 to construct multiple receptor conformations^59^, ^60^ representative of the response of the binding site residues to the template ligand AS-605240. This generated a set of 18 models. Figure 4 c shows results of the best three models (models 7, 10 and 17) obtained from the induced fit docking run of PI3Kα. Of the generated models, only one structure (model 7) showed a very modest improvement in enrichment (Table 2; table S5 in the Supporting Information).

A homology model of PI3Kα based upon the ZSTK474–PI3K*δ* structure (PDB: 2WXL) was built using Prime 2.2^61^ followed by manual realignment of some residues. This model was then used for rigid receptor docking experiments and as a starting point for successive generations of models made by induced fit docking with AS-605240. Figure 4 d shows ROC curves for the initial homology model and derived structures. This homology model is noticeably better than the 2RD0 crystal structure in discriminating between active compounds and decoys. The induced fit docking models were better again. Two induced fit models, model 3 and model 5 of the induced fit structures showed much improved enrichment (Table 2; table S5 in the Supporting Information). Although ultimately neither the homology model nor induced fit structures outperform the parent PI3K*δ* structure, 2WXL; this induced fit modeling approach is clearly useful for the development of homology models with good ability to discriminate active compounds from compounds in the decoy set. However, it should be noted that docking lacked the discriminatory power to predict the relative potencies of the test thiazolidinedione or rhodanine compounds.

**Figure 8:**
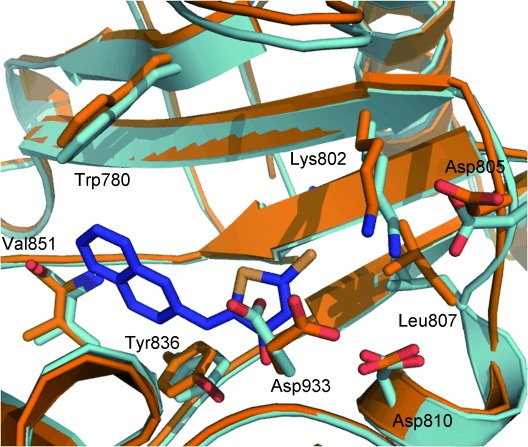
Alignment of model 3 (pale blue) and model 5 (orange) highlighting differences observed in side-chain conformations of Asp 933, Asp 805, Leu 807 and Lys 802 important for inhibitor binding.

**Figure 9:**
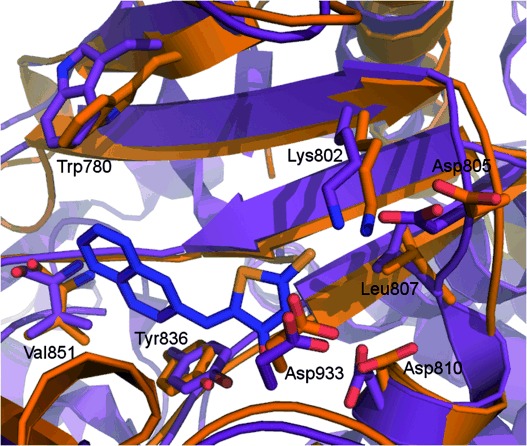
Alignment of 2RD0 (purple) and model 5 (orange) highlighting differences observed in side chain Asp 805, Leu 807 and Lys 802 conformations important for inhibitor binding.

A structural comparison of the induced fit models with the PI3K*δ* structure show a close backbone alignment with most variation observed in side-chain orientation (Figures 8 and 9). Measurements show preserved interactions consistent with those observed for AS-605240 in the PI3Kγ X-ray structure.

The crystal structure of apo-PI3Kα and model 5 show Asp 933 closely overlaid, but marked differences are apparent around Asp 805, Leu 807 and Lys 802 (Figure 9). Overall, the variation observed in side-chain conformations of key residues provides an explanation for the poor performance of the apo-PI3Kα structure.^62^ The apparent success of the apo-PI3Kα in docking studies against other molecules, such as PIK75, may reflect a reduced importance of those residues in binding that molecule compared to the relatively small and flat arylidene thiazolidinediones.

It is also important to note that subtle changes in side-chain orientation of active site residues yielded significant changes in the docking results, both in terms of enrichment and also in the observed binding poses of compounds. While model 3 predicted the majority of compounds to bind thiazolidinediones in an analogous pose to the X-ray structures, model 5 accommodated ligands in a flipped pose as shown in Figure 7. Residues Lys 802, Tyr 836, Trp 780 and Asp 810, which are situated within 5 Å of bound ligand (Figure 8), contribute to this, but of most importance appears to be Asp 933, which in model 5 is not well placed for binding with the thiazolidinedione ring.

The synthesis and assay of thiazolidinedione derivatives have been widely used in medicinal chemistry research. They have been described positively as “privileged scaffolds”^63^ or negatively as “frequent hitters” or pan-assay interference compounds (PAINS).^64^, ^65^ As well as inhibiting PI3K, thiazolidinedione derivatives are clinically used PPARγ agonists (pioglitazone, rosiglitazone) and aldose reductase inhibitors (epalrestat), and have research applications as antibacterial, antimalarial, anti-inflammatory, antiviral, herbicidal, insecticidal, antifungal, anticancer, anthelmintic agents, and for the treatment of Alzheimer’s disease, central nervous systems (CNS) disorders, diabetes, cardiovascular, cystic fibrosis and thrombocytopenia.^63^, ^66^ They bind to targets as diverse as G protein-coupled receptor 40 (GPR40),^67^ protein tyrosine phosphatase 3 (PRL-3),^68^ cyclooxygenase 2 (COX-2),^69^ the peptidoglycan biosynthesis enzymes, MurB, MurC and MurG,^63^ B cell lymphoma-2 (Bcl-2),^70^ phosphodiesterase 4 (PDE4),^71^ fungal protein mannosyl transferase 1 (PMT1),^72^ tumor necrosis factor alpha (TNF-α),^73^ hepatitis C virus nonstructural protein 3 (HCV NS3) and NS5b polymerase (HCV NS5b),^74^, ^75^ cytosolic phospholipase A2α (cPLA2α),^76^ proto-oncogene serine/threonine protein kinase (Pim-1),^77^ cyclin-dependent kinase 2 (CDK2),^54^ HIV-1 integrase,^78^ serotonin N-acetyltransferase (AANAT),^79^ and glycogen synthase kinase-3β (GSK-3β).^80^ Several of the most potent compounds identified here have also been picked up in other screening campaigns, offering a cautionary note to the possibility of off-target effects. Frequent reporting in their identification via screening may be due to the low IC_50_ activity assigned for a hit, which is often ∼25 μm, representing only weak affinity for the target of interest. On the other hand, the drug-like properties of low-molecular weight, low log *P*, presence of both hydrogen-bond donors and acceptors, and the capacity for multiple approaches to structural elaboration recommend them as small molecules in a fragment-based screening approach, and the perceived limitations could be addressed by structural modification.

## Conclusions

In summary, having noted the report of PI3Kγ thiazolidinedione inhibitors in the literature, we devised a broadened library aiming to discover inhibitors targeting the PI3Kα isoform and 12 inhibitors with sub-micromolar IC_50_ values were identified. We attempted in silico docking experiments and showed that the active compounds could be readily identified from decoy compounds. Docking results were improved by using higher resolution structures and liganded structures of the PI3Kγ and PI3Kδ isoforms, which perform better than the apo form of the actual target, PI3Kα. It was interesting that, in this case, homology of the protein to the target was less important than the presence of a ligand in the binding site or resolution of the structure chosen. Improved enrichment using a PI3Kα structure was observed with the use of induced fit virtual screening experiments for a PI3Kα homology model, rather than the apo structure. The homology models derived from induced fit docking studies showed that specific conformers surrounding key residues markedly influence the docking result. As a validation of the use of virtual screening in this context, it is apparent that, with the correct selection of protein model, most of the potent inhibitors could be identified from the decoy set. With a reliable model of PI3Kα in hand, docking could be well utilized in future screening campaigns for isoform selective compounds.

## Experimental Section

### Chemistry

All chemical reagents acquired from Sigma–Aldrich and Fluka were used without further purification, while compounds **40**–**42**, **44**–**47** and **49**–**73** (figure S1 in the Supporting Information) were acquired from Maybridge (Thermo Fisher Scientific Inc., USA). Experimental data on synthesized compounds is provided in the Supporting Information.

### Computational modeling

PI3K X-ray structures (2RD0, 2A5U, 2WXL) were obtained from the PDB (http://www.rcsb.org). All solvents and small molecules were removed from structures. Protein preparation and refinement was performed using Maestro 9.0 or 9.1 Protein Preparation Wizard, and default parameters were used to optimize protein structures. Receptor grid generation was confined to 20 Å from the binding site ligand. Alignment of X-ray structures 2A5U and 2RD0 in Maestro was performed to determine the 2RD0 binding site. Ligands were constructed in Sybyl-X, energy minimized using the Tripos force field default settings for 1000 steps, imported into Maestro and prepared using LigPrep 2.3. Adjustment of protonation state was performed manually in Maestro. Docking calculations were performed in Glide 5.5 or 5.6 using extra precision (XP) mode only. Sampling was limited to 10 000 ligand poses per docking run and only one pose per ligand was retained. The set of 1000 drug-like decoy compounds, with an average molecular weight of 360, was obtained from Schrödinger (http://www.schrodinger.com). The decoy set enriched with our seventy-three compounds was docked into each X-ray structure and ranked by GlideScore.

Homology models of PI3Kα were built using the PI3K*δ* (2WXL)^25^ crystal structure as the template. The structure was edited to 378 amino acids encompassing the catalytic domain only. Human PI3Kα and *mus musculus* PI3K*δ* sequences were obtained from the US National Center for Biotechnology Information and aligned using Protein BLAST (http://www.ncbi.nlm.nih.gov/).^81^ Homology models were generated in Prime (version 2.2) with selected loops refined using extended sampling, then minimized. Induced fit docking utilizing Prime (version 2.2) and Glide (version 5.6) XP mode was performed using default settings unless otherwise specified. Serono compound AS-605240 was docked initially for identification of optimal model protein structures. Receptor-grid generation for each of the nine selected structures was prepared as described above. Docking calculations used XP mode for the nine structures using the Schrödinger decoy set enriched with our 73 compounds and ranked by GlideScore. ROC curves were generated using Microsoft Excel. Images were created using PyMOL.^82^ PI3Kα models 3 and 5 in pdb format with sets of 73 docked ligands for each of these models in sdf format are provided in the Supporting Information.

### Inhibition Assay

PI3K protein was purified from cell lysates of transfected Sf9 insect cells and diluted in assay buffer (10 mm HEPES, 25 mm NaCl, 0.125 μg mL^−1^ bovine serum albumin (BSA), 2 mm basal medium Eagle (BME), final concentrations) containing 2.5 μg each of L-α-phosphatidylinositol and 1,2-diacyl-*sn*-glycero-3-phospho-L-serine in 96-well plates. Inhibitors were dissolved and diluted in DMSO. Reactions were started upon addition of 10 μm ATP with 40 μCi mL^−1^ γ-^32^P-ATP (Perkin–Elmer) and 2 mm MgCl_2_ (final concentrations). Reactions were incubated for 2 h at room temperature, terminated upon addition of 2 n HCl. Lipids were extracted in CHCl_3_/CH_3_OH (1:1). Extracted organic fractions containing ^32^P-PI(3)P were quantitated via addition of Microscint C using a TopCount 96-well plate scintillation counter measuring counts per minute (cpm). Prism 5 (GraphPad Software) was used to calculate IC_50_ values and inhibition curves (Table 1; see also the Supporting Information).

## Notes

Detailed facts of importance to specialist readers are published as ”Supporting Information”. Such documents are peer-reviewed, but not copy-edited or typeset. They are made available as submitted by the authors.
