# Supplementary material for: Thiazolidinedione-Based PI3Kα Inhibitors: An Analysis of Biochemical and Virtual Screening Methods
Source: ChemMedChem. 2011 Jan 4;6(3):514–22. doi: 10.1002/cmdc.201000467 (PMC3187668; doi:10.1002/cmdc.201000467)
Supplement: Supplementary file 1 [file cmdc0006-0514-sd1.pdf]

CHEMMEDCHEM
Chemistry & Drug Discovery

Supporting Information

© Copyright Wiley-VCH Verlag GmbH & Co. KGaA, 69451 Weinheim, 2011

Thiazolidinedione-Based PI3Kα Inhibitors: An Analysis of Biochemical and Virtual Screening Methods

Jo-Anne Pinson,[a] Oleg Schmidt-Kittler,[b] Jiuxiang Zhu,[b] Ian G. Jennings,[a]
Kenneth W. Kinzler,[b] Bert Vogelstein,[b] David K. Chalmers,[a] and Philip E. Thompson*[a]

cmdc_201000467_sm_miscellaneous_information.pdf

Supplementary data for Pinson et al

18

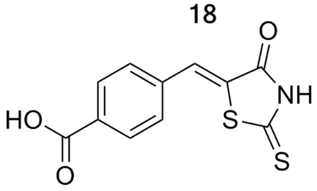

21

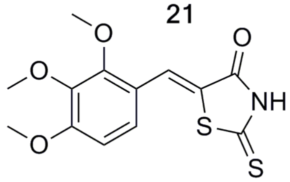

22

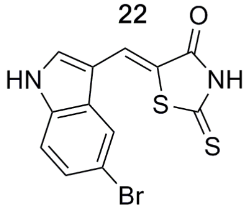

23

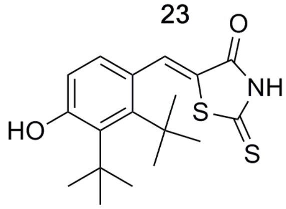

24

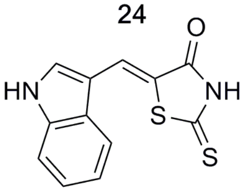

25

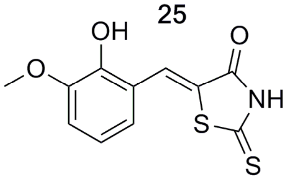

26

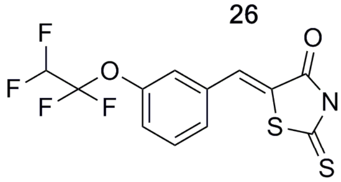

27

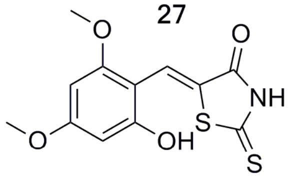

28

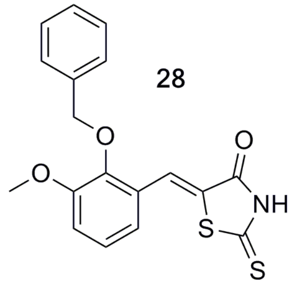

29

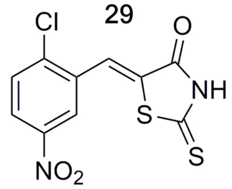

30

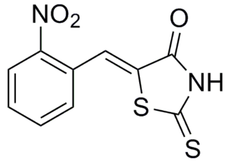

31

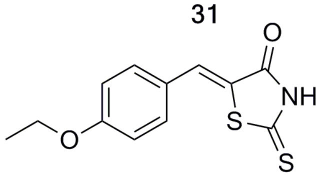

32

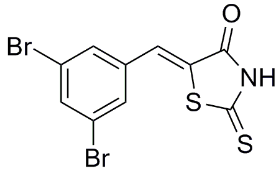

33

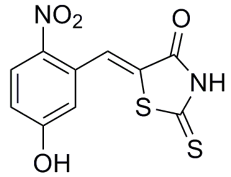

34

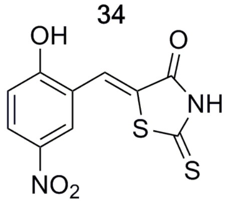

35

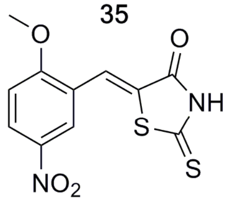

36

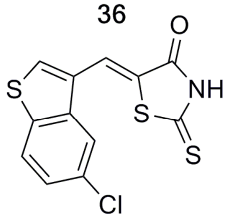

37

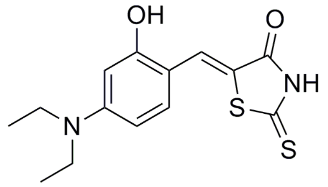

38

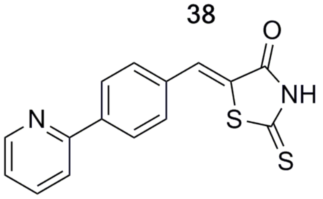

39

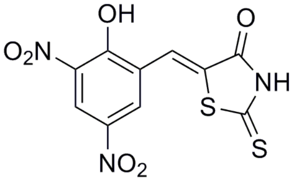

Figure S1. Compound structures not described in main text.

48

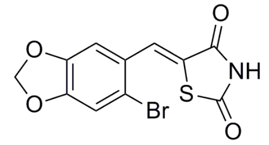

49

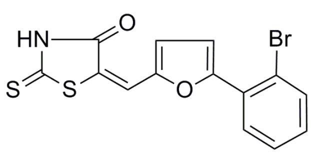

50

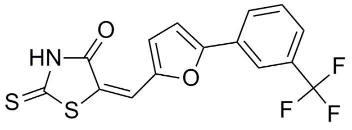

51

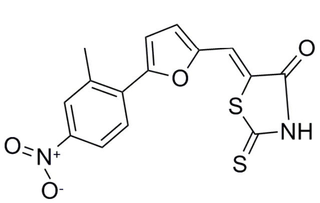

52

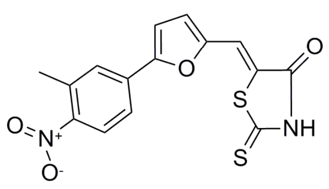

53

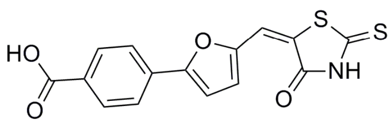

54

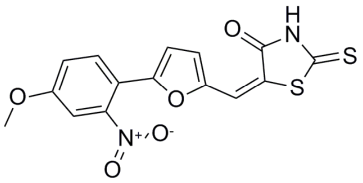

55

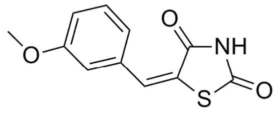

56

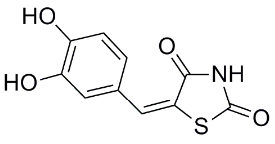

57

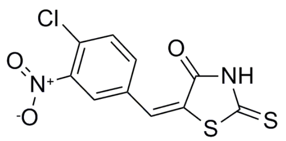

58

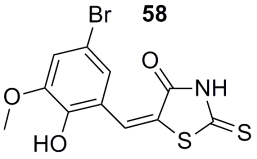

59

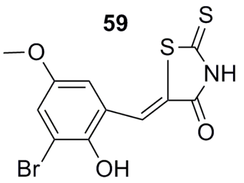

60

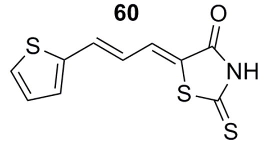

61

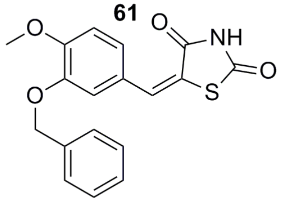

62

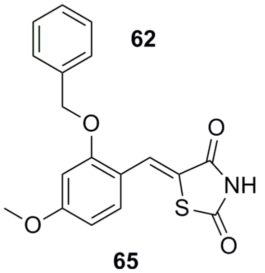

63

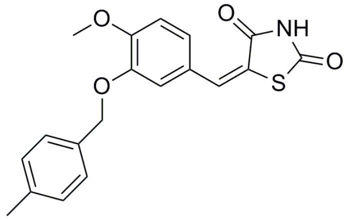

64

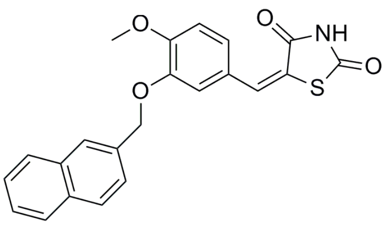

65

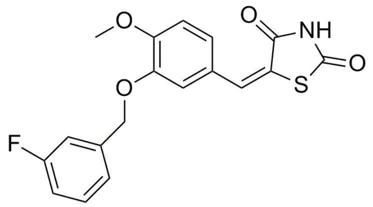

66

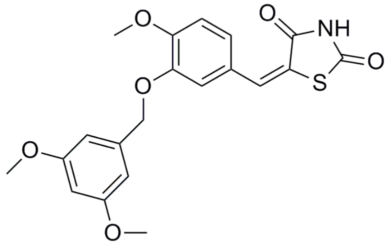

67

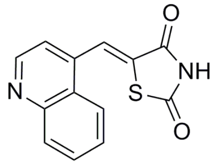

68

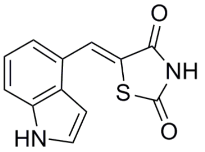

Figure S1 cont.. Compound structures not described in main text.

69

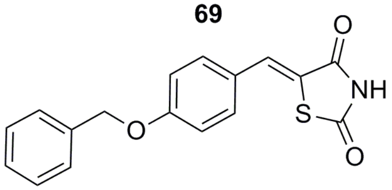

70

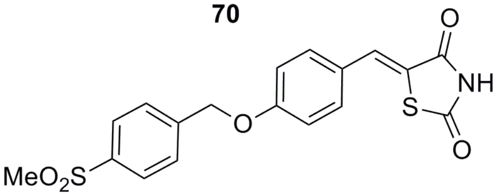

71

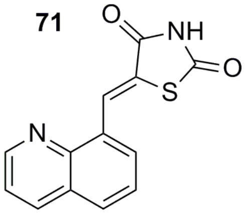

72

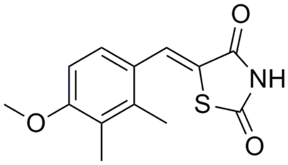

73

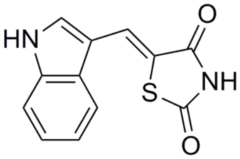

Figure S1 cont.. Compound structures not described in main text.

Compound #	IC50 v. PI3Kα	IC50 v. PI3Kγ	α/γ	Compound #	IC50 v. PI3Kα	IC50 v. PI3Kγ	α/γ
1 AS604850	4.5	0.25	18	38	>100	>100	1.0
2 AS605240	0.060	0.0080	7.5	39	14	9.2	1.5
3	0.050	0.040	1.3	40	3.0	.41	7.3
4	0.27	0.11	2.5	41	11	1.4	7.9
5	.45	.12	3.8	42	0.14	0.060	2.3
6	50	>100	<0.50	43	8.7	>100	<0.087
7	1.9	1.0	1.9	44	9.0	>100	<0.090
8	7.3	27	0.27	45	0.40	0.20	2.0
9	4.4	9.3	0.47	46	4.0	3.5	1.1
10	2.7	4.9	0.55	47	0.80	1.0	0.80
11	0.069	0.042	1.6	48	33	70	0.47
12	0.15	0.12	1.2	49	6.0	6.0	1.
13	2.7	0.25	11	50	6.0	8.0	0.75
14	11	1.5	7.1	51	0.70	0.70	1.0
15	9.4	3.1	3.0	52	4.0	3.5	1.1
16	86	4.0	22	53	15	9.5	1.6
17	22	1.4	16	54	14	10	1.4
18	>100	>100	1.0	55	1.0	1.0	1.0
19	.0028	.0013	2.2	56	3.5	1.2	2.9
20	8.3	.62	13	57	7.5	2.5	3.0
21	68	>100	<0.68	58	2.6	4.0	0.65
22	15	15	1	59	1.2	2.0	0.60
23	30	24	1.3	60	3.7	3.5	1.1
24	>100	>100	1.0	61	50	>100	<0.5
25	>100	>100	1.0	62	>100	>100	1.0
26	14	14	1.0	63	>100	>100	1.0
27	11	9.2	1.2	64	>100	>100	1.0
28	>100	>100	1.0	65	>100	>100	1.0
29	42	16	2.6	66	>100	>100	1.0
30	>100	>100	1.0	67	1.8	1.0	1.8
31	5.4	9.1	0.59	68	>100	>100	1.0
32	>100	>100	1.0	69	>100	>100	1.0
33	7.2	4.9	1.5	70	>100	>100	1.0
34	23	6.7	3.4	71	>100	>100	1.0
35	28	13	2.2	72	20	26	0.77
36	30	11	2.6	73	>100	>100	1.0
37	64	>100	<0.64				

Table S1 IC50 data for inhibition of PI3Kα and PI3Kγ

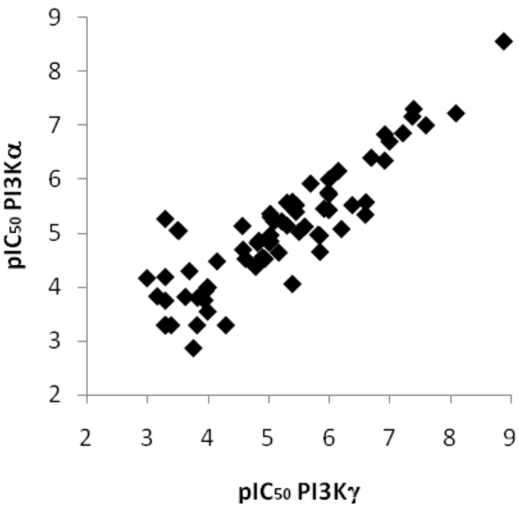

Figure S2. Plot of pIC_{50} values for PI3Kα versus γ isoforms.

Compound	Estimated pKa	Compound	Estimated pKa
**3**	7.53	**24**	8.16
**8**	8.44	**25**	7.37
**9**	7.68	**26**	7.33
**10**	7.80	**27**	NR *
**11**	8.01	**28**	7.34
**12**	7.95	**29**	6.77
**13**	7.72	**30**	7.22
**14**	6.42	**31**	7.83
**15**	7.11	**32**	6.98
**16**	7.07	**33**	7.60
**17**	6.98	**34**	7.11
**18**	7.60	**35**	6.86
**19**	7.08	**36**	7.46
**20**	7.53	**37**	8.04
**21**	7.55	**38**	7.46
**22**	7.56	**39**	6.54
**23**	7.98		

Table S2. pKa values calculated using ACD Chemsketch for thirty-three synthesized rhodanines. * Compound failed using two versions of software, thus likely not considered weakly acidic.

Compound # (protonated)	G-Score (2rd0)	G-Score (2a5u)	G-Score (2wxl)	Compound # (protonated)	G-Score (2rd0)	G-Score (2a5u)	G-Score (2wxl)
1 AS604850	-5.95	-9.34	-7.79	38	-5.77	-7.95	-6.97
2 AS605240	-8.10	-7.59	-8.03	39	-6.22	-5.39	-7.63
3	-6.95	-8.50	-9.41	40	-5.84	-7.54	-8.34
4	-6.64	-9.06	-9.71	41	-6.86	-8.10	-6.69
5	-6.86	-8.44	-8.53	42	-6.47	-7.31	-8.85
6	-7.20	-6.03	-10.21	43	-6.55	-6.57	-9.64
7	-7.20	-7.42	-9.60	44	-5.23	-7.18	-7.60
8	-5.92	-10.15	-6.07	45	-2.96	-6.31	-8.32
9	-5.47	-8.11	-8.42	46	-5.36	-6.93	-7.56
10	-6.49	-7.49	-8.08	47	-6.41	-9.69	-10.47
11	-5.60	-8.24	-7.11	48	-7.60	-6.76	-8.65
12	-5.50	-8.20	-8.95	49	-6.43	-7.21	-8.04
13	-8.32	-7.54	-9.82	50	-6.48	-6.88	-8.60
14	-5.88	-6.27	-6.98	51	-5.58	-7.22	-7.76
15	-5.54	-6.74	-8.76	52	-5.00	-6.50	-8.12
16	-6.66	-5.81	-8.38	53	-6.32	-7.78	-10.09
17	-6.67	-7.71	-7.68	54	-6.18	-7.71	-7.91
18	-7.16	-9.31	-8.75	55	-6.02	-8.24	-8.03
19	-7.56	-6.37	-8.19	56	-6.68	-10.13	-11.73
20	-6.53	-5.96	-9.01	57	-6.63	-8.85	-8.22
21	-7.02	-4.25	-7.81	58	-6.61	-5.43	-6.96
22	-6.33	-8.21	-8.30	59	-7.12	-5.37	-6.97
23	-6.53	-3.75	-3.57	60	-5.99	-5.46	-6.35
24	-7.08	-7.41	-7.31	61	-7.19	-5.47	-8.56
25	-6.91	-5.65	-7.80	62	-7.19	-7.31	-9.72
26	-7.09	-8.01	-8.50	63	-6.23	-6.09	-9.55
27	-5.76	-7.92	-7.97	64	-6.86	-7.50	-9.80
28	-6.95	-5.83	-5.90	65	-7.41	-5.92	-9.19
29	-5.68	-7.19	-5.85	66	-7.42	-8.90	-9.66
30	-6.42	-6.54	-6.80	67	-5.64	-7.85	-7.94
31	-6.60	-7.51	-8.32	68	-7.33	-6.60	-7.99
32	-6.17	-8.48	-6.44	69	-7.03	-6.90	-9.05
33	-6.47	-6.69	-6.69	70	-5.71	-8.11	-8.93
34	-5.24	-7.50	-7.94	71	-6.12	-7.03	-8.59
35	-5.60	-5.15	-6.97	72	-6.38	-5.58	-7.73
36	-6.10	-7.75	-8.48	73	-5.54	-7.62	-8.58
37	-5.44	-8.11	-6.38				

Table S3. GlideScore (G-Score) docking results of 73 protonated compounds for X-ray structures 2rd0, 2a5u and 2wxl.

Compound # (deprotonated)	G-Score (2rd0)	G-Score (2a5u)	G-Score (2wxl)	Compound # (deprotonated)	G-Score (2rd0)	G-Score (2a5u)	G-Score (2wxl)
1 AS604850	-7.23	-7.30	-8.23	38	-4.86	-6.61	-6.17
2 AS605240	-8.38	-6.16	-7.08	39	-4.83	-2.81	-4.93
3	-7.41	-7.18	-8.79	40	-2.87	-5.62	-8.11
4	-6.60	-6.82	-9.62	41	-5.25	-6.88	-6.62
5	-6.53	-6.87	-9.64	42	-5.50	-3.45	-8.27
6	-5.75	-7.71	-8.89	43	-5.85	-7.16	-4.97
7	-7.49	-6.82	-9.50	44	-3.97	-5.82	-6.93
8	-5.63	-8.66	-9.21	45	-4.92	-6.73	-7.42
9	-5.80	-6.97	-6.57	46	-7.78	-6.21	-5.59
10	-5.64	-6.20	-7.26	47	-5.63	-7.47	-8.60
11	-5.47	-7.35	-7.13	48	-5.75	-6.32	-7.98
12	-5.36	-7.24	-8.22	49	-5.66	-6.11	-7.66
13	-6.81	-8.43	-7.00	50	-5.09	-6.53	-8.47
14	-3.97	-3.71	-6.40	51	-5.17	-6.60	-7.08-
15	-6.43	-6.13	-9.19	52	-4.99	-6.74	-6.84
16	-6.01	-4.07	-7.57	53	-6.32	-8.12	-9.46
17	-6.29	-6.62	-6.13	54	-5.52	-5.37	-6.73
18	-6.13	-7.71	-10.10	55	-5.79	-6.52	-7.27
19	-8.36	-7.30	-7.99	56	-5.78	-5.55	-8.36
20	-8.02	-7.32	-7.65	57	-6.41	-6.46	-8.09
21	-6.68	-3.61	-3.99	58	-5.74	-7.82	-6.76
22	-5.20	-8.19	-6.71	59	-5.59	-5.09	-6.05
23	-3.71	-4.29	-3.74	60	-5.60	-5.64	-7.43
24	-4.96	-8.04	-6.79	61	-6.20	-6.95	-7.53
25	-5.99	-6.89	-5.68	62	-5.96	-6.87	-9.32
26	-5.39	-4.88	-7.76	63	-6.53	-6.13	-6.38
27	-5.56	-6.78	-7.36	64	-6.26	-6.91	-4.98
28	-5.11	-5.74	-7.32	65	-5.79	-6.09	-7.72
29	-4.99	-5.07	-6.20	66	-6.04	-7.55	-7.60
30	-3.70	-5.33	-6.27	67	-4.83	-6.21	-8.03
31	-6.20	-6.13	-8.06	68	-6.08	-3.55	-6.46
32	-5.42	-4.64	-6.70	69	-5.95	-6.37	-4.85
33	-5.62	-5.56	-7.35	70	-5.66	-7.09	-7.66
34	-4.76	-6.38	-6.38	71	-5.19	-5.81	-8.41
35	-3.89	-4.99	-5.57	72	-5.94	-5.18	-7.16
36	-7.17	-6.61	-6.97	73	-5.05	-7.87	-7.77
37	-5.07	-6.47	-6.08				

Table S4. GlideScore (G-Score) docking results of 73 deprotonated compounds for X-ray structures 2rd0, 2a5u and 2wxl.

Compound # protonated	G-Score (2rd0 IFD)	G-Score (2wxl model)	G-Score (model 3)	G-Score (model 5)	Compound # protonated	G-Score (2rd0 IFD)	G-Score (2wxl model)	G-Score (model 3)	G-Score (model 5)
1 AS604850	-8.36	-9.33	-10.38	-10.03	38	-7.25	-9.44	-8.15	-9.93
2 AS605240	-8.51	-10.19	-10.37	-10.95	39	-7.83	-9.48	-7.28	-10.47
3	-7.66	-10.74	-10.83	-10.23	40	-9.31	-9.17	-9.95	-11.35
4	-9.39	-11.66	-9.65	-10.52	41	-7.80	-8.03	-9.70	-10.70
5	-6.61	-10.60	-10.93	-9.77	42	-7.73	-9.23	-9.89	-10.56
6	-8.18	-11.57	-12.41	-8.94	43	-8.12	-10.37	-10.65	-11.18
7	-9.57	-11.65	-10.54	-10.71	44	-8.99	-9.55	-11.25	-12.21
8	-8.27	-9.65	-10.97	-10.91	45	-7.85	-10.31	-7.58	-8.94
9	-7.89	-8.96	-9.41	-9.94	46	-8.21	-9.39	-8.42	-11.96
10	-7.75	-9.29	-9.69	-9.99	47	-8.66	-12.42	-10.74	-11.59
11	-9.08	-10.07	-4.86	-10.52	48	-7.47	-10.84	-8.03	-8.87
12	-9.44	-11.25	-10.45	-10.40	49	-7.72	-10.10	-9.54	-12.15
13	-8.99	-11.53	-11.19	-12.02	50	-8.68	-10.68	-9.83	-11.27
14	-7.67	-9.05	-9.49	-9.85	51	-7.97	-8.22	-9.19	-9.70
15	-9.27	-10.20	-8.46	-10.31	52	-8.48	-8.58	-9.34	-9.29
16	-7.52	-8.92	-8.67	-10.19	53	-8.55	-10.09	-9.12	-11.14
17	-8.13	-8.05	-7.79	-9.76	54	-7.52	-7.14	-7.94	-11.45
18	-8.86	-11.01	-10.39	-11.28	55	-8.72	-9.09	-9.90	-10.50
19	-8.49	-8.01	-11.53	-10.98	56	-9.16	-13.69	-10.20	-12.35
20	-6.98	-9.78	-9.60	-10.33	57	-7.90	-8.86	-9.79	-10.80
21	-8.24	-6.95	-7.96	-9.85	58	-8.12	-8.71	-7.23	-11.12
22	-8.33	-8.17	-10.84	-11.36	59	-8.19	-8.60	-7.73	-10.26
23	-5.82	-7.56	-3.84	-6.67	60	-7.62	-8.76	-8.24	-9.55
24	-8.83	-9.89	-10.59	-10.83	61	-8.30	-10.69	-10.59	-10.74
25	-7.99	-8.44	-9.12	-10.88	62	-8.26	-8.94	-11.32	-10.64
26	-9.12	-10.50	-9.26	-11.56	63	-8.54	-9.14	-10.78	-11.35
27	-5.85	-8.28	-8.00	-9.07	64	-8.22	-9.57	-10.98	-11.18
28	-7.85	-9.49	-9.05	-11.93	65	-8.56	-9.22	-10.73	-11.39
29	-7.69	-8.29	-9.76	-9.88	66	-8.93	-10.08	-11.00	-11.80
30	-7.73	-8.51	-8.84	-9.78	67	-9.24	-9.23	-10.03	-11.36
31	-7.30	-8.75	-8.29	-10.00	68	-7.54	-10.07	-9.65	-10.22
32	-9.23	-9.03	-7.39	-10.83	69	-7.95	-11.29	-10.53	-9.80
33	-7.44	-8.62	-7.76	-9.13	70	-8.34	-9.65	-11.20	-11.28
34	-7.24	-8.02	-8.14	-10.04	71	-8.21	-10.04	-10.44	-10.87
35	-7.29	-8.18	-7.88	-9.09	72	-7.80	-9.61	-10.07	-9.75
36	-7.78	-9.18	-9.69	-11.25	73	-8.95	-9.74	-9.42	-10.82
37	-7.83	-8.41	-8.09	-10.09					

Table S5. GlideScore (G-Score) docking results of 73 protonated compounds for homology model, 2rd0 induced fit and homology model induced fit.

Experimental

^{1}H-NMR spectra were recorded with either a 300 MHz Varian widebore NMR spectrometer or a 400 MHz Bruker Ultrashield-Avance III NMR spectrometer. ^{13}C-NMR spectra were recorded with either a 600 MHz Varian Oxford AS600-Unity Inova NMR spectrometer or a 400 MHz Bruker Ultrashield-Avance III NMR spectrometer. Results were recorded as follows: chemical shift values are expressed δ units acquired in DMSO (2.50 ppm) as reference, multiplicity (s = singlet, d = doublet, t = triplet, q = quartet, m = multiplet), integration and coupling constants (*J*) in Hertz. Mass spectra were acquired in the positive and negative mode using electrospray ionization obtained on a Micromass Platform II single quadrupole mass spectrometer equipped with an atmospheric pressure (ESI/APCI) ion source with sample management facilitated by an Agilent 1100 series HPLC system using MassLynx version 3.5 software. High Resolution Mass Spectrometry analyses were collected on a Waters Micromass LCT Premier XE Orthogonal Acceleration Time-of-Flight Mass Spectrometer coupled to an Alliance 2795 Separation Module using MassLynx version 4.1 software. Melting point determination was performed uncorrected using a Mettler Toledo MP50 melting point apparatus.

Synthesis: A mixture of the aldehyde (1 eq), rhodanine or thiazolidinedione (2 eq), β-alanine (2 eq), in glacial acetic acid was heated at 100 °C for 3 hr. Upon cooling, the product was isolated by filtration and washed with glacial acetic acid and water. If required, purification via crystallization as described.

(**Z)-5-(benzo[d][1,3]dioxol-5-ylmethylene)thiazolidine-2,4-dione (3)**: 99% yellow powder. mp: 246-249 °C (lit. 247-249 °C)[1]; ^{1}H-NMR (300 MHz, [D$_6$]DMSO), δ 12.5 (br s, 1H), 7.71 (s, 1H), 7.14 (m, 3H), 6.13 (s, 2H); ESI-MS, *m/z* 248.1 $[M-H]^-$.

(Z)-5-(benzo[d][1,3]dioxol-5-ylmethylene)-2-thioxothiazolidin-4-one (4) : 100% orange crystals; mp: 247 °C (dec.) (lit. 246-250 °C)[2]; ^{1}H-NMR (300 MHz, [D$_6$]DMSO), δ 13.7 (br s, 1H), 7.58 (s, 1H), 7.14 (m, 3H), 6.14 (s, 2H); ESI-MS, *m/z* 266.1 $[M+H]^+$.

(Z)-5-(benzo[d][1,3]dioxol-5-ylmethylene)-4-thioxothiazolidin-2-one (5): 100% red crystals; mp: 264 °C (dec.); ^{1}H-NMR (300 MHz, [D$_6$]DMSO), δ 13.9 (br s, 1H), 8.04 (s, 1H), 7.27 (d, *J* = 8.1 Hz, 1H), 7.21 (s, 1H), 7.10 (d , *J* = 8.2 Hz, 1H), 6.15 (s, 2H); ^{13}C (75 MHz, [D$_6$]DMSO), δ 195.14, 170.63, 150.02, 148.45, 136.43, 127.74, 127.63, 127.31, 109.46, 109.46, 102.33; ESI-MS, *m/z* 264.1 $[M-H]^-$.

(**Z)-5-(benzo[d][1,3]dioxol-5-ylmethylene)imidazolidine-2,4-dione (6)**: 93% off-white powder; mp 239-242 °C; ^{1}H-NMR (300 MHz, [D$_6$]DMSO), δ 13.7 (br s, 1H), 7.27 (d, *J* = 1.7 Hz, 1H), 7.13 (dd, *J* = 8.4, 1.8 Hz, 1H), 6.95 (d, *J* = 8.1 Hz, 1H), 6.36 (s, 1H), 6.07 (s, 2H); ESI-MS, *m/z* 233.1 $[M+H]^+$; HRMS (ESI): $[M-H]^-$ 231.0421 observed 231.0411 $[M-H]^-$ calculated.

(Z)-5-(benzo[d][1,3]dioxol-5-ylmethylene)thiazolidine-2,4-dithione (7): 98% purple powder; mp: 230 °C (dec.) (MeCN/H_2O, 4:1); ^{1}H-NMR (300 MHz, [D$_6$]DMSO), δ 7.78 (s, 1H), 7.29 (d, *J* = 8.2 Hz, 1H), 7.23 (s, 1H), 7.14 (d, *J* = 8.2 Hz, 1H), 6.17 (s, 2H); ^{13}C NMR (100 MHz, [D$_6$]DMSO), δ 196.68, 195.89, 150.39, 148.58, 135.60, 132.16, 128.03, 127.85, 109.61, 109.56, 102.41; ; HRMS (ESI): $[M-H]^-$ 279.9586 observed 279.9566 $[M-H]^-$ calculated

(Z)-5-(2,3-dihydroxybenzylidene)-2-thioxothiazolidin-4-one (8): 59% brown needles; mp: 225-227 °C; ^{1}H-NMR (300 MHz, [D$_6$]DMSO), δ 13.73 (br s, 1H), 9.91 (s, 1H), 9.58 (s, 1H), 7.87 (s, 1H), 6.93 (dd, *J* = 3.3Hz, 1H), 6.79 (d, *J* = 2.0 Hz, 1H), 6.78 (s, 1H); ^{13}C NMR (100 MHz, [D$_6$]DMSO), δ 196.15, 169.56, 146.38, 145.90, 127.74, 123.75, 120.58, 119.88, 119.15, 117.94; HRMS (ESI): 253.9924 $[M+H]^+$ observed 253.9940 $[M+H]^+$calculated.

(Z)-5-(2-hydroxy-4-methoxybenzylidene)-2-thioxothiazolidin-4-one (9): 75% brown crystals; mp: 229 °C (dec.); ^{1}H-NMR (300 MHz, [D$_6$]DMSO), δ 13.63 (br s, 1H), 10.83 (s,1H), 7.81 (s,1H), 7.26 (d, *J* = 8.8 Hz, 1H), 6.59 (dd, *J* = 8.8, 2.5 Hz,1H), 6.51 (d, *J* = 2.5 Hz,1H), 3.78 (s, 3H); ^{13}C NMR (150 MHz, [D$_6$]DMSO), δ 195.77, 169.62, 163.28, 159.55, 130.95, 127.54, 120.29, 113.20, 107.12, 101.13, 55.45; HRMS (ESI): 265.9953 [M-H]$^-$ observed 265.9951 [M-H]$^-$ calculated .

(Z)-5-(4-(allyloxy)benzylidene)-2-thioxothiazolidin-4-one (10): 81% green crystals; mp: 197-199 °C; ^{1}H-NMR (300 MHz, [D$_6$]DMSO), δ 13.72 (br s, 1H), 7.61 (s, 1H), 7.57 (d, *J* = 9.0 Hz, 2H), 7.13 (d, *J* = 9.0 Hz, 2H), 6.06 (m, 1H), 5.36 (ddq, *J* = 33.9, 10.5, 1.5 Hz, 2H), 4.67 (dt, *J* = 5.2, 1.5 Hz, 2H); HRMS (ESI): 276.0137 [M-H]$^-$ observed 276.0158 [M-H]$^-$ calculated.

(Z)-5-(4-hydroxy-3,5-dimethylbenzylidene)-2-thioxothiazolidin-4-one (11): 87% green crystals; mp: 282-286 °C; ^{1}H-NMR (300 MHz, [D$_6$]DMSO), δ 13.7 (br s, 1H), 9.31 (s, 1H), 7.49 (s, 1H), 7.22 (s, 2H), 2.23 (s, 6H); HRMS (ESI): 264.0164 [M-H]$^-$ observed 264.0158 [M-H]$^-$ calculated.

(Z)-5-(4-hydroxy-3-methylbenzylidene)-2-thioxothiazolidin-4-one (12): 92% yellow powder; mp: 296-298 °C; ^{1}H-NMR (300 MHz, [D$_6$]DMSO), δ 13.7 (br s, 1H), 10.36 (s, 1H), 7.51 (s, 1H), 7.32 (dd, *J* = 4.5, 12 Hz,2H), 6.94 (d, *J* = 9 Hz, 1H), 2.15 (s, 3H); ^{13}C NMR (150 MHz, [D$_6$]DMSO), δ 195.58, 169.49, 158.75, 133.69, 132.71, 130.66, 125.53, 123.86, 120.63, 115.64, 15.90; HRMS (ESI): 250.0002 [M-H]$^-$ observed 250.0002 [M-H]$^-$ calculated.

(Z)-5-(3-hydroxy-4-methoxybenzylidene)-2-thioxothiazolidin-4-one (13): 100% yellow solid; mp: 225-227 °C; ^{1}H-NMR (300 MHz, [D$_6$]DMSO), δ 13.74 (br s, 1H), 9.58 (s, 1H), 7.52 (s, 1H), 7.12 (d, *J* = 1.8 Hz, 1H), 7.10 (s, 1H), 7.03 (d, *J* = 1.8 Hz, 1H), 3.84 (s, 3H); HRMS (ESI): 266.4987 [M-H]$^-$ observed 266.4988 [M-H]$^-$ calculated..

(Z)-5-((2,6-dichloropyridin-3-yl)methylene)-2-thioxothiazolidin-4-one (14): 957% yellow needles; mp: 226-228 °C; ^{1}H-NMR (300 MHz, [D$_6$]DMSO), δ 11.95 (br s, 1H), 8.00 (d, *J* = 7.9 Hz, 1H), 7.72 (d, *J* = 7.9 Hz,1H), 7.56 (s, 1H); ^{13}C NMR (100 MHz, [D$_6$]DMSO), δ 194.95, 172.04, 169.03, 149.88, 149.55, 140.59, 131.81, 127.40, 124.56, 123.26; HRMS (ESI): 288.9000 [M-H]$^-$ observed 288.9069 [M-H]$^-$ calculated.

(Z)-5-(pyridin-3-ylmethylene)-2-thioxothiazolidin-4-one (15): 91% yellow powder; mp: 291-293 °C; ^{1}H-NMR (300 MHz, [D$_6$]DMSO), δ 8.84 (d, *J* = 2.3 Hz, 1H), 8.64 (dd, *J* = 4.8, 1.5 Hz, 1H), 7.95 (dt, *J* = 8, 1.9 Hz, 1H), 7.68 (s, 1H), 7.57 (dd, *J* = 8.1, 4.8 Hz, 1H); ^{13}C NMR (100 MHz, [D$_6$]DMSO) δ 195.50, 169.39, 151.84, 150.87, 136.34, 129.16, 128.20, 127.98, 124.54; HRMS (ESI): 220.9848 [M-H]$^-$ observed 220.9849 [M-H]$^-$ calculated.

(Z)-4-((4-oxo-2-thioxothiazolidin-5-ylidene)methyl)benzonitrile (16): 89% yellow powder ; mp: 286 °C (dec.); ^{1}H-NMR (300 MHz, [D$_6$]DMSO), δ 7.99 (d, *J* = 8.4 Hz, 2H), 7.78 (d, *J* = 8.3 Hz, 2H), 7.70 (s, 1H); ^{13}C NMR (100 MHz, [D$_6$]DMSO), δ 195.24, 169.24, 137.34, 133.07, 133.05, 130.79, 130.77, 129.17, 129.15, 118.37, 112.20; HRMS (ESI): 244.9844 [M-H]$^-$ observed 244.9849 [M-H]$^-$ calculated.

(Z)-5-(4-nitrobenzylidene)-2-thioxothiazolidin-4-one (17) : 88% yellow needles; mp: 252-255 °C; ^{1}H-NMR (300 MHz, [D$_6$]DMSO), δ 8.34 (d, *J* = 8.8 Hz, 2H), 7.87 (d, *J* = 8.8 Hz, 2H), 7.75 (s, 1H); HRMS (ESI): 264.9747 [M-H]$^-$ observed 264.9753 [M-H]$^-$ calculated.

(Z)-4-((4-oxo-2-thioxothiazolidin-5-ylidene)methyl)benzoic acid (18): 93% yellow powder; mp: >300 °C; ^{1}H-NMR (300 MHz, [D$_6$]DMSO), δ 13.19 (br s, 1H), 8.06 (d, *J* = 8.3 Hz, 2H), 7.73 (s, 1H), 7.69 (d, *J* = 4.5 Hz, 2H); HRMS (ESI): 263.9796 [M-H]$^-$ observed 263.9795 [M-H]$^-$ calculated.

(Z)-5-(quinoxalin-6-ylmethylene)-2-thioxothiazolidin-4-one (19): 88% brown crystals; mp: 297 °C (dec.); ^{1}H-NMR (300 MHz, [D$_6$]DMSO), δ 9.03 (d, *J* = 3.3Hz, 2H), 8.32 (s, 1H), 8.23 (d, *J* = 8.7Hz, 1H), 8.05 (d, *J* = 8.7Hz, 1H), 7.91 (s, 1H); HRMS (ESI): 271.9967 [M-H]$^-$ observed 271.9958 [M-H]$^-$ calculated.

(Z)-5-((2,2-difluorobenzo[d][1,3]dioxol-5-yl)methylene)-2-thioxothiazolidin-4-one (20): 84% yellow powder; mp: 181-183 °C; ^{1}H-NMR (300 MHz, [D$_6$]DMSO), δ 11.90 (br s, 1H), 7.64 (d, *J* = 1.5 Hz, 1H), 7.58 (s, 1H), 7.57 (d, *J* = 8.4 Hz, 1H), 7.45 (dd, *J* = 8.7, 1.8 Hz, 1H); HRMS (ESI): 299.9619 [M-H]$^-$ observed 299.9606 [M-H]$^-$ calculated.

(Z)-2-thioxo-5-(2,3,4-trimethoxybenzylidene)thiazolidin-4-one (21): 95% yellow solid; mp: 206-209 °C (MeOH); ^{1}H-NMR (300 MHz, [D$_6$]DMSO), δ 13.70 (br s, 1H), 7.67 (s, 1H), 7.17 (d, *J* = 8.8 Hz, 1H), 7.01 (d, *J* = 8.9 Hz, 1H), 3.87 (s, 6H), 3.77 (s, 3H); HRMS (ESI): 310.02192 [M-H]$^-$ observed 310.0213 [M-H]$^-$ calculated.

(Z)-5-((5-bromo-1H-indol-3-yl)methylene)-2-thioxothiazolidin-4-one (22): 84% red powder; mp: >300 °C; ^{1}H-NMR (300 MHz, [D$_6$]DMSO), δ 13.58 (s, 1H), 12.43 (s, 1H), 8.22 (d, *J* = 1.7 Hz, 1H), 7.95 (s, 1H), 7.85 (d, *J* = 3.0 Hz, 1H), 7.40 (m, 2H); HRMS (ESI): 336.9102 [M-H]$^-$ observed 336.9110[M-H]$^-$ calculated.

(Z)-5-(2,3-di-tert-butyl-4-hydroxybenzylidene)-2-thioxothiazolidin-4-one (23): 53% orange needles; mp: 256-257 °C (MeOH); ^{1}H-NMR (300 MHz, [D$_6$]DMSO), δ 13.73 (br s, 1H), 7.91 (s, 1H), 7.63 (s, 1H), 7.37 (s, 2H), 1.41 (s, 18H); ^{13}C NMR (150 MHz, [D$_6$]DMSO), δ 195.46, 169.38, 157.10, 139.51, 133.62, 128.14, 128.12, 124.35, 124.34, 121.01, 34.63, 29.96; HRMS (ESI): 348.1104 [M-H]$^-$ observed 348.1097 [M-H]$^-$ calculated.

(Z)-5-((1H-indol-3-yl)methylene)-2-thioxothiazolidin-4-one (24): 91% orange needles; mp: 285-289 °C; ^{1}H-NMR (300 MHz, [D$_6$]DMSO), δ 13.57 (br s, 1H), 12.32 (s, 1H), 7.95 (s, 1H), 7.93 (s, 1H), 7.83 (d, *J* = 3.0 Hz, 1H), 7.51 (d, *J* = 7.3 Hz, 1H) 7.24 (m, 2H); HRMS (ESI): 259.0013 [M-H]$^-$ observed 259.0006 [M-H]$^-$ calculated.

(Z)-5-(2-hydroxy-3-methoxybenzylidene)-2-thioxothiazolidin-4-one (25): 88% yellow needles; mp: 244 °C (dec.); ^{1}H-NMR (300 MHz, [D$_6$]DMSO), δ 9.88 (s, 1H), 7.86 (s, 1H), 7.11 (dd, *J* = 6.4, 3.1 Hz, 1H), 6.93 (d, *J* = 6.8 Hz, 1H), 6.91 (s, 1H), 3.82 (s, 3H); ESI-MS, *m/z* 266.3 [M-H]$^-$.

(Z)-5-(3-(1,1,2,2-tetrafluoroethoxy)benzylidene)-2-thioxothiazolidin-4-one (26): 77% yellow crystals; mp: 189-191 °C (MeOH); ^{1}H-NMR (300 MHz, $CDCl_3$), δ 9.28 (s, 1H), 7.62 (s, 1H), 7.52 (dd, *J*=11.8, 4.8 Hz, 1H), 7.40 (d, *J*=7.9 Hz, 1H), 7.33 (s, 1H), 7.32 (d, *J*=6.9 Hz, 1H), 5.95 (tt, *J*=53, 2.8 Hz); ^{13}C NMR (100 MHz, [D$_6$]DMSO) δ 195.51, 169.35, 148.85, 135.38, 135.06, 131.43, 130.01, 128.57, 127.37, 126.93, 123.56, 123.24 (*J* = 240 Hz); HRMS (ESI): 335.9771 [M-H]$^-$ observed 335.9782 [M-H]$^-$ calculated.

(Z)-5-(2-hydroxy-4,6-dimethoxybenzylidene)-2-thioxothiazolidin-4-one (27): 80% brown crystals; mp: 239-242 °C; ^{1}H-NMR (300 MHz, [D$_6$]DMSO), δ 13.34 (br s, 1H), 10.96 (s, 1H), 7.86 (s, 1H), 6.15 (d, *J* = 6.3 Hz, 2H), 3.86 (s, 3H), 3.79 (s, 3H); ^{13}C NMR (150 MHz, [D$_6$]DMSO), δ 197.47, 172.03, 169.96, 164.60, 159.96, 126.74, 121.11, 102.85, 93.61, 90.34, 55.52, 55.49; ESI-MS, *m/z* 296.2 [M-H]$^-$.

(Z)-5-(2-(benzyloxy)-3-methoxybenzylidene)-2-thioxothiazolidin-4-one (28): 82% yellow powder; mp: 182-184 °C; ^{1}H-NMR (300 MHz, [D$_6$]DMSO), δ 7.67 (s, 1H), 7.30 (ddd, *J* = 25.5, 5.8, 4 Hz, 7H), 6.95 (dd, *J* = 6.4, 2.7 Hz, 1H), 5.03 (s, 2H), 3.90 (s, 3H); ^{13}C NMR (100 MHz, [D$_6$]DMSO) δ 195.94, 169.10, 152.89, 146.77, 136.54, 128.67, 128.34, 128.16, 127.41, 126.75, 126.36, 125.07, 119.84, 115.56, 75.01, 56.01; HRMS (ESI): 356.0406 [M-H]$^-$ observed 356.0421 [M-H]$^-$ calculated.

(Z)-5-(2-chloro-5-nitrobenzylidene)-2-thioxothiazolidin-4-one (29): 79% yellow needles; mp: 250-252 °C; ^{1}H-NMR (300 MHz, [D$_6$]DMSO), δ 8.29 (dd, *J* = 2.7, 8.7 Hz, 1H), 8.27 (d, *J* = 2.4 Hz, 1H), 7.95 (d, *J* = 8.7 Hz, 1H), 7.68 (s, 1H); ^{13}C NMR (100 MHz, [D$_6$]DMSO) δ 194.92, 169.38, 146.73, 140.68, 132.48,

132.42, 131.81, 125.93, 123.86, 123.56; HRMS (ESI): 298.9353 $[M-H]^-$ observed 298.9357 $[M-H]^-$ calculated.

(**Z)-5-(2-nitrobenzylidene)-2-thioxothiazolidin-4-one (30)**: 100% yellow crystals; mp: 197-199 °C; ^{1}H-NMR (300 MHz, [D_6]DMSO), δ 11.90 (br s, 1H), 8.20 (d, *J* = 8.1 Hz, 1H), 7.87 (d, *J* = 9.2 Hz, 2H), 7.72 (dd, *J* =10.9, 7.8 Hz, 2H); HRMS (ESI): 264.9737 $[M-H]^-$ observed 264.9747 $[M-H]^-$ calculated.

(Z)-5-(4-ethoxybenzylidene)-2-thioxothiazolidin-4-one (31): 100% yellow crystals; mp: 225-227 °C; ^{1}H-NMR (300 MHz, [D_6]DMSO), δ 13.70 (br s, 1H), 11.90 (br s, 1H), 7.61 (s, 1H), 7.56 (d, *J* = 9.0 Hz,2H), 7.10 (d, *J* = 8.7 Hz, 2H), 4.11 (q, *J* = 7.0 Hz, 2H), 1.34 (t, *J* = 7.0 Hz, 3H); HRMS (ESI): 264.0158 $[M-H]^-$ observed 264.0165 $[M-H]^-$ calculated.

(Z)-5-(3,5-dibromobenzylidene)-2-thioxothiazolidin-4-one (32): 93% yellow crystals; mp: 276-278 °C; ^{1}H-NMR (300 MHz, [D_6]DMSO), δ 7.98 (t, *J* = 1.7 Hz, 1H), 7.78 (d, *J* = 1.6 Hz, 2H), 7.60 (s, 1H); HRMS (ESI): 375.8107 $[M-H]^-$ observed 375.8124 $[M-H]^-$ calculated.

(Z)-5-(5-hydroxy-2-nitrobenzylidene)-2-thioxothiazolidin-4-one (33): 66% green needles; mp: 225 °C (decomp.); ^{1}H-NMR (300 MHz, [D_6]DMSO), δ 13.80 (br s, 1H), 11.31 (s, 1H), 8.16 (d, *J* = 8.8 Hz, 1H), 7.91 (s, 1H), 7.07-6.95 (m, 2H); HRMS (ESI): 280.9696 $[M-H]^-$ observed 280.9686 $[M-H]^-$ calculated.

(Z)-5-(2-hydroxy-5-nitrobenzylidene)-2-thioxothiazolidin-4-one (34): 100% brown crystals; mp: 253 °C (EtOH); ^{1}H-NMR (300 MHz, [D_6]DMSO), δ 8.20 (m, 2H), 7.74 (s, 1H), 7.12 (d, *J* = 9.0 Hz, 1H); ^{13}C NMR (100 MHz, [D_6]DMSO), δ 195.28, 169.27, 162.82, 127.75, 127.11, 124.99, 124.57, 120.39, 116.63; HRMS (ESI): 280.9684 $[M-H]^-$ observed 280.9686 $[M-H]^-$ calculated.

(Z)-5-(2-methoxy-5-nitrobenzylidene)-2-thioxothiazolidin-4-one (35): 96% orange crystals; mp: 251-254 °C; ^{1}H-NMR (300 MHz, [D_6]DMSO), δ 8.37 (dd, *J* = 9.2, 2.8 Hz, 1H), 8.22 (d, *J* = 2.7 Hz, 1H), 7.71 (s, 1H), 7.38 (d, *J* = 9.3 Hz, 1H), 4.06 (s, 3H); ^{13}C NMR (100 MHz, [D_6]DMSO), δ 195.21, 169.14, 162.42, 140.79, 128.42, 127.81, 124.64, 123.97, 121.98, 112.65, 57.11; HRMS (ESI): 294.9839 $[M-H]^-$ observed 294.9853 $[M-H]^-$ calculated.

(Z)-5-((5-chlorobenzo[b]thiophen-3-yl)methylene)-2-thioxothiazolidin-4-one (36): 96% yellow solid; mp: 297-299 °C; ^{1}H-NMR (300 MHz, [D_6]DMSO), δ 13.85 (br s, 1H), 8.32 (d, *J* = 1.9 Hz, 1H), 8.22 (s, 1H), 8.16 (d, *J* = 8.7 Hz, 1H), 7.92 (s, 1H), 7.54 (dd, *J* = 8.6, 1.9 Hz, 1H); HRMS (ESI): 309.9220 $[M-H]^-$ observed 309.9227 $[M-H]^-$ calculated.

(Z)-5-(4-(diethylamino)-2-hydroxybenzylidene)-2-thioxothiazolidin-4-one (37): 100% red powder; mp: 277 °C (dec.); ^{1}H-NMR (300 MHz, [D_6]DMSO), δ 13.60 (br s, 1H), 7.52 (s, 1H), 7.43 (d, *J* = 9.0 Hz, 2H), 6.83 (d, *J* = 9.0 Hz, 2H), 4.05 (s, 3H), 3.04 (d, *J* = 2.9 Hz, 7H); ESI-MS, *m/z* 307.2 $[M-H]^-$.

(Z)-5-(4-(pyridin-2-yl)benzylidene)-2-thioxothiazolidin-4-one (38): 81% yellow needles; mp: 264-266 °C; ^{1}H-NMR (300 MHz, [D_6]DMSO), δ 8.71 (d, *J* = 3.8 Hz, 1H), 8.26 (d, *J* = 8.4 Hz, 2H), 8.07 (d, *J* = 8. 0 Hz, 1H), 7.92 (td, *J* = 7.8, 1.7 Hz, 1H), 7.73 (d, *J* = 8.4 Hz, 2H), 7.70 (s, 1H), 7.41 (dd, *J* = 7.2, 4.8 Hz, 1H); ^{13}C NMR (150 MHz, [D_6]DMSO) δ 195.47, 169.42, 154.55, 149.75, 140.22, 137.38, 133.21, 131.24, 130.93, 127.32, 126.22, 125.90, 123.08, 120.85; HRMS (ESI): 299.0307 $[M-H]^-$ observed 299.0307 $[M-H]^-$ calculated.

(Z)-5-(2-hydroxy-3,5-dinitrobenzylidene)-2-thioxothiazolidin-4-one (39): 44% red crystals; mp: 243-245 °C; ^{1}H-NMR (300 MHz, [D_6]DMSO), δ 14.20 (br s, 1H), 9.19 (s, 1H), 8.77 (s, 1H), 8.22 (s, 1H); HRMS (ESI): 325.9550 $[M-H]^-$ observed 325.9547 $[M-H]^-$ calculated.

References:

[1] W. H. Burton, W. L. Budde, C. C. Cheng, *J.Med.Chem* **1970**, *13*, 1009.

[2] K. Bourahla, A. Derdour, M. Rahmouni, F. Carreaux, J. P. Bazureau, *Tet. Let.* **2007**, *48*, 5785.
